# Les tumeurs malignes de type glandes salivaires primitives du poumon: étude clinico-pathologique de 10 cas

**DOI:** 10.11604/pamj.2022.43.206.29691

**Published:** 2022-12-23

**Authors:** Ahlem Bchir, Slim Ben Ahmed, Moncef Mokni

**Affiliations:** 1 Laboratoire d´Anatomie et Cytologie Pathologique, Centre Hospitalier Universitaire Farhat Hached de Sousse, Sousse, Tunisie,; 2 Faculté de Médecine Ibn Jazzar, Sousse, Tunisie,; 3 Service d´Oncologie Médicale, Centre Hospitalier Universitaire Farhat Hached de Sousse, Sousse, Tunisie

**Keywords:** Tumeur de type glande salivaire, carcinome adénoïde kystique, carcinome muco-épidermoïde, poumon, pronostic, Salivary gland-type tumor, adenoid cystic carcinoma, mucoepidermoid carcinoma, lung, prognosis

## Abstract

Les tumeurs malignes primitives de type glandes salivaires pulmonaires sont rares, leurs présentation clinico-pathologique est particulière, touchant le sujet jeune, non associées au tabac et de siège proximal. Pour une prise en charge optimale, il est important de les distinguer des autres tumeurs broncho-pulmonaires, dont elles ne partagent ni la biologie ni le traitement ni l´évolution. Notre étude était rétrospective, incluant tous les tumeurs malignes de type glandes salivaires primitives du poumon, sur une période de 32 ans, allant du Janvier 1987 au décembre 2019. Nous avons colligé 10 cas: cinq hommes et cinq femmes, avec un sex-ratio à 1. L´âge moyen de nos patients était de 47,4 ans. Les examens radiologiques montrent une tumeur nodulaire de siège proximal dans tous les cas dont la taille variait de 1 à 5,8 cm. Histologiquement, les 10 cas correspondaient à 6 cas de carcinome muco-épidermoïde. Tous les cas avaient un stade localisé ou localement avancé, à l´exception d´un cas de carcinome muco-épidermoïde qui était métastatique d´emblée. Les tumeurs pulmonaires de type glandes salivaires forment un groupe hétérogène de tumeurs peu agressives mais associées à une fréquence élevée de récidive et de métastases tardives imposant une surveillance à long terme.

## Abstract (en)

Primary pulmonary salivary gland-type tumors are rare and characterized by a specific clinico-pathological manifestation. They are proximal tumors affecting young subjects and not associated with smoking. For optimal management, it is important to distinguish them from other bronchopulmonary tumors, with which they don´t share either biology or treatment and outcome. We conducted a retrospective study, including all primary malignant salivary gland-type tumors of the lung, over a period of 32 years, from January 1987 to December 2019. We collected data from the medical records of 10 patients: 5 men and 5 women (sex ratio 1). The average age of our patients was 47.4 years. In all cases radiological examinations showed proximally located nodular tumor ranging in size from 1 to 5.8 cm. Histological examination objectified mucoepidermoid carcinoma (6 out of 10 cases). All patients had localized or locally advanced tumors, with the exception of one case of metastatic mucoepidermoid carcinoma. Pulmonary salivary gland-type tumors correspond to a heterogeneous group of tumours. They are not very aggressive but are associated with a high frequency of recurrences and late metastases, requiring long-term monitoring.

## Introduction

Les tumeurs malignes primitives de type glandes salivaires (TMPGS) représentent moins de 1% de tous les cancers broncho-pulmonaires. Elles sont représentées par le carcinome adénoïde kystique (CAK), le carcinome muco-épidermoïde (CME), le carcinome épithélial-myoépithélial (CEM) et le carcinome ex-adénome pléomorphe. Elles sont issues des glandes péries bronchiques et sont le plus souvent de localisation proximale. Leur traitement est essentiellement chirurgical. Du point de vue étiopathogénique, elles ne sont pas associées au tabagisme ou à d´autres facteurs de risque contrairement aux autres tumeurs broncho-pulmonaires. Pour une prise en charge optimale, il est important de les distinguer des autres tumeurs broncho-pulmonaires, dont elles ne partagent ni la biologie ni le traitement ni l´évolution [1]. L´objectif de ce travail est de décrire les aspects clinico-pathologiques de 10 cas de tumeurs malignes primitives de type glandes salivaires du poumon colligés sur une période de 32 ans avec revue de littérature.

## Méthodes

**Type et lieu d´étude**: il s´agit d´une étude rétrospective et descriptive dans le cadre d´une thèse de docteur en médecine portant sur 10 cas de TMPGS pulmonaires, colligés au service d´Anatomie et de Cytologie pathologiques du CHU Farhat Hached de Sousse, Tunisie avec une période de 32 ans, du 1 janvier 1987 au 31 décembre 2019.

**Population cible**: les critères d´inclusion sont tous les patients porteurs de TMPGS d´origine primitive au niveau du poumon, dont le diagnostic a été porté dans le service d´Anatomie et de Cytologie Pathologiques au CHU Farhat Hached de sousse, Tunisie. Les critères d´exclusion sont la présence de métastase pulmonaire d´un CAK dont le primitif était au niveau des glandes salivaires et les cas dont les dossiers étaient inexploitables.

**Collecte des données**: la collecte des données cliniques, para cliniques et évolutives a été faite à partir des dossiers des patients et des fiches d´anatomopathologie. Pour tous les cas, les données suivantes ont été relevées: a) l´âge et le sexe; b) les antécédents; c) le délai diagnostique; d) les circonstances de découverte; e) l´examen physique avec l´évaluation de l´état général en utilisant le score de l´OMS; f) les résultats de la fibroscopie (siège, aspect macroscopique); g) examens radiologique (radiographie du thorax, scanner thoracique avec injection de produit de contraste); h) l´appareil utilisé pour l´exploration fonctionnelle respiratoire est: Vmax version 21-1A/Viasys heathcare; i) les données thérapeutiques: le type de chirurgie, les suites opératoires et les traitements adjuvants; j) le stade cTNM: 8^e^ édition; k) les données évolutives: le suivi des patients, les récidives et la survie avec un recul de 5 ans.

**Démarche diagnostique**: le diagnostic a été posé sur: a) des biopsies (7 cas/10); b) les pièces opératoires (7 cas/10); c) les étalements cytologiques (4 cas/10). Une relecture des lames histologiques à l´hématoxyline éosine et des lames de l´étude en immunohistochimie (IHC) a été faite.

**Instruments de mesure**: la classification des tumeurs, la stadification pTNM et le grading adoptés étaient ceux de l‘Organisation Mondiale de la Santé (OMS 2015). Les caractéristiques des anticorps (Ac) utilisés sont rapportées dans le Tableau 1. Le marquage est classé semi quantitativement en 4 scores selon le pourcentage des cellules marquées, il est: a) négatif: si moins de 5% des cellules sont marquées; b) focal: si 5% à 9% des cellules sont marquées; c) modéré: si 10% à 30% des cellules sont marquées; d) diffus: si plus que 30% des cellules sont marquées. On n´a pas recouru à des méthodes statistiques vu que c´est une étude de cas de série et que notre série se limite à 10 cas seulement.

**Tableau 1:** caractéristiques des anticorps utilisés dans notre étude

Variables	Clone	Code	Dilution	Source	Marquage
carcinome adénoïde kystique (CAK)	AE1/AE3	M630	1/50	DAKO	membranaire
CD117	Rb	A4502	1/400	DAKO	membranaire
P63	7JUL	M7247	1/25	Novo	nucléaire
				castra	
actine muscle lisse (AML)	1A4	M0851	1/50	DAKO	cytoplasmique
PS100	Rbpoly	Z0311	1/300	DAKO	cytoplasmique
Syn	27G12	M776	1/100	Novo	cytoplasmique
				castra	
Chr A	5H7	M869	1/100	Novo	cytoplasmique
				castra	
Antigène Carcino Embryonnaire (ACE)	11-7	M7072	1/25	DAKO	cytoplasmique
Calponine	CALP	M3556	1/50	DAKO	cytoplasmique
Ki67	MIB-1	M722	1/50	DAKO	nucleaire
cytokératine 7 (CK7)	RN7	M7018	1/50	Novo	membranaire
				castra	
antigène épithélial membranaire (EMA)	E29	M0613	1/100	DAKO	membranaire
NSE	BBS/NC/H14	M873	1/100	DAKO	cytoplasmique
Vim	V9	M725	1/100	DAKO	cytoplasmique

**Consentement des patients**: les auteurs déclarent qu´ils ont reçu un consentement écrit par tous les patients qui ont participé à cette étude.

## Résultats

**Caractéristiques de la population étudiée**: la population globale étudiée est constituée de 10 cas de TMPGS. L´âge de nos patients variait entre 20 et 69 ans avec un âge moyen de 47,4 ans et le sex ratio était de 1. Pour le CAK, l´âge variait de 30 et 69 ans avec un âge moyen de 55,7 ans et le sex ratio était de 1/3. Pour le CME, l´âge variait entre 20 et 69 ans avec un âge moyen de 41,8 ans et le sex ratio était de 1/2. Des antécédents de broncho-pneumopathie chronique obstructive ont été notés dans un cas et de bronchite chronique dans deux cas. Le tabagisme a été retrouvé dans quatre cas. Un tabagisme passif avec une exposition à la fumée de bois a été retrouvé dans un cas. L´alcoolisme a été retrouvé dans deux cas.

**Figure 1:**
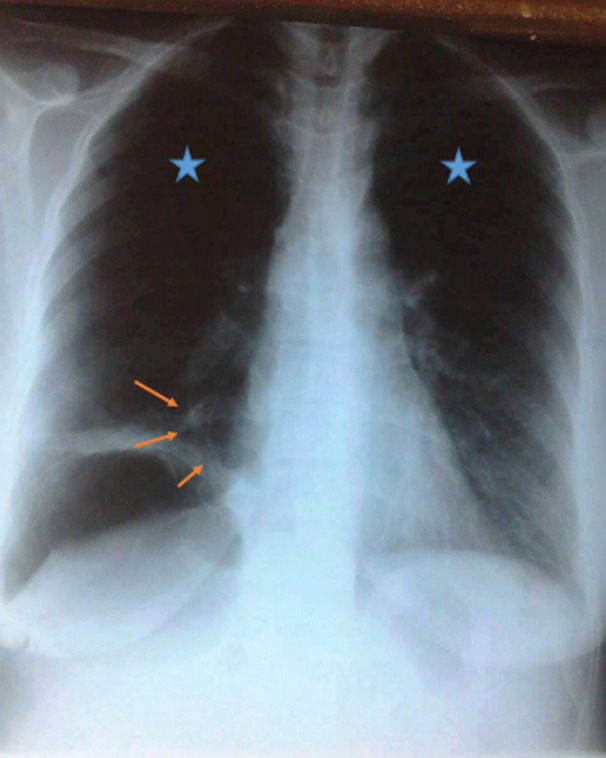
radiographie du thorax: atélectasie du lobe inférieur droit (au niveau de la flèche orange) avec emphysème obstructif (étoile bleue)

**Etude analytique**: la symptomatologie la plus fréquente était l´association d´une toux avec douleur basithoracique, suivie par l´hémoptysie, notée dans deux cas. Les autres symptomatologies étaient une hémoptysie dans deux cas, une toux sèche dans un cas, une toux avec expectorations dans deux cas, une douleur basithoracique avec fièvre dans un cas. Aucun patient n´avait présenté de syndrome cave supérieur. Le délai moyen entre le début des symptômes et la confirmation du diagnostic était de 7,45 mois, ce délai variait entre un mois et trois ans. Pour le CAK, il variait de 3 mois à 6 mois. Pour le CME, ce délai variait de 1 mois à 3 ans. L´examen physique était sans particularités. Cinq patients avaient un score OMS à 1, 4 un score OMS à 2 et un un score OMS à 3. La radiographie thoracique, montrait une opacité de localisation paracardiaque dans trois cas et périphérique dans quatre cas. L´opacité était rétractile dans deux cas. Il existait également une atélectasie avec emphysème obstructif dans un cas (Figure 1).

**Figure 2:**
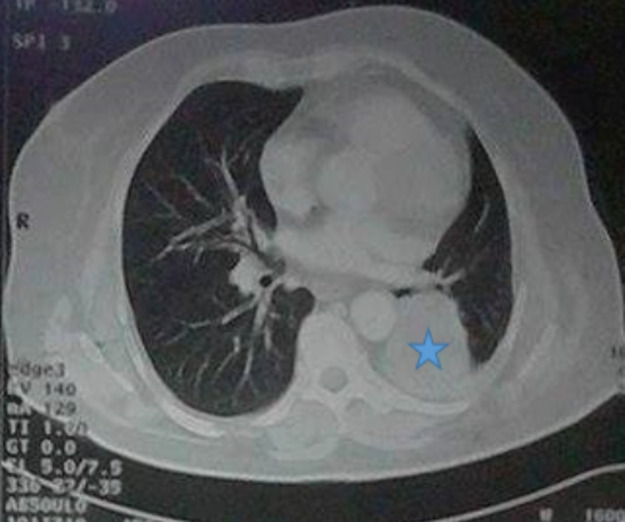
TDM thoracique montrant une masse lobaire inférieure (étoile bleue)

Le scanner thoracique montrait un nodule ou une masse tumorale, de siège proximal dans tous les cas : intéressant la bronche souche dans quatre cas, la bronche lobaire dans quatre autres et de siège hilaire dans un cas. L´aspect tumoral était tissulaire dans un cas, partiellement calcifié dans un cas, et nécrosé dans un autre cas. La taille moyenne de la tumeur était de 3,6 cm avec des extrêmes de 1 à 5,8cm. La tumeur envahissait le médiastin moyen dans un cas, englobait les structures vasculaires dans 1 autre et était responsable d´un collapsus du lobe inférieur dans deux cas. Des adénopathies hilaires et médiatisnales étaient notées dans deux cas (Figure 2).

**Figure 3:**
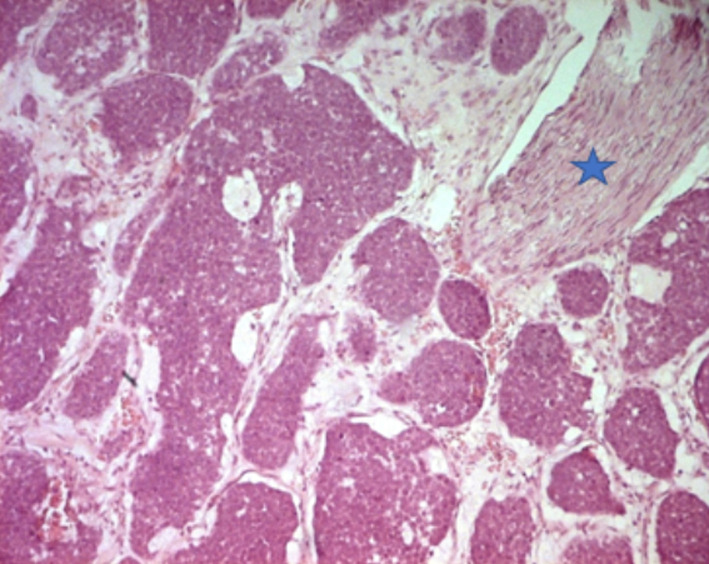
carcinome adénoïde kystique avec engainement périnerveux (étoile bleue) (HE x 100)

**Figure 4:**
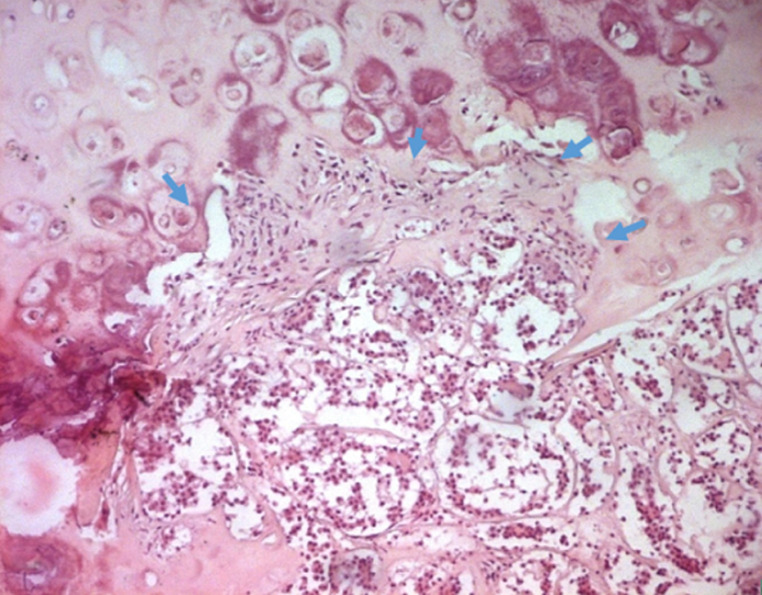
carcinome adénoïde kystique, infiltration du cartilage bronchique (flèche bleue) (HE x 100)

La fibroscopie bronchique montrait un aspect bourgeonnant dans les 10 cas, dont un était polypoïde pédiculé. La tumeur était saignante au contact dans cinq cas et vascularisée dans deux cas. Il y avait une obstruction bronchique dans quatre cas. Une infiltration de la carène était notée dans un cas de CAK. Le bilan d´extension comportait un scanner abdominal, réalisé dans les 10 cas, montrant une métastase surrénalienne dans un cas de CME. Pour le stade clinique, cinq patients avaient un stade localisé (trois stade I et deux stade II), quatre patients avaient un stade localement avancé (stade III). Un patient avait un stade métastatique (stade IV). Le diagnostic positif a été obtenu par une biopsie endoscopique dans quatre cas et par thoracotomie dans six cas (Tableau 2). Le traitement chirurgical, effectué chez sept patients consistait en une pneumonectomie chez trois patients, une lobectomie chez deux patients, une bilobectomie chez un patient et une sleeve résection chez un autre patient. Un curage ganglionnaire a été effectué chez six patients. A l´étude anatomo-pathologique, l´examen macroscopique a révélé une tumeur de siège proximal, bourgeonnante et nodulaire dans les sept cas. La taille moyenne était de 4 cm, avec des extrêmes de 2 à 7 cm, la tumeur était de consistance ferme, de couleur beige ou blanc grisâtre, comportant des foyers hémorragiques dans un cas, le CME avait en plus un aspect mucoïde et microkystique, à la coupe. Histologiquement, les 10 cas de TMPGS étaient répartis en six cas de CME et quatre cas de CAK. Leurs aspects microscopiques étaient similaires à leurs contre parties observés au niveau des glandes salivaires. Le diagnostic de carcinome adénoïde kystique était posé devant une prolifération tumorale organisée en massifs cribriformes ou tube à contenu éosinophile, positif avec la coloration de l´acide périodique de Schiff (PAS). Les cellules étaient monomorphes, cubiques ou arrondies, à cytoplasme parfois clair et réduit. Les atypies étaient discrètes et les mitoses étaient peu fréquentes. Des engainements périnerveux étaient fréquemment notés. Une infiltration de la trachée était notée dans un cas (Figure 3, Figure 4).

**Tableau 2:** modalité du diagnostif positif

Mode de diagnostic	Biopsie endoscopique	Thoracotomie
carcinome adénoīde kystique (CAK)	3	1
carcinome muco-épidermoïde(CME)	1	5
**Total**	4	6

**Figure 5:**
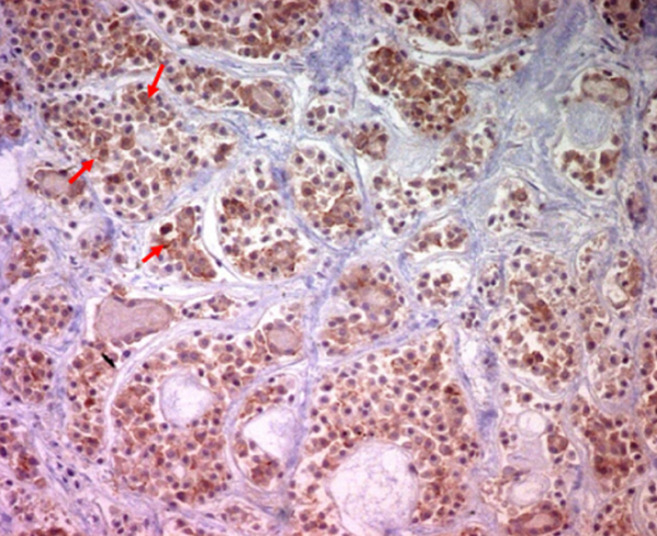
carcinome adénoïde kystique, expression du CD 117 (flèche rouge) (IHC x 100)

**Figure 6:**
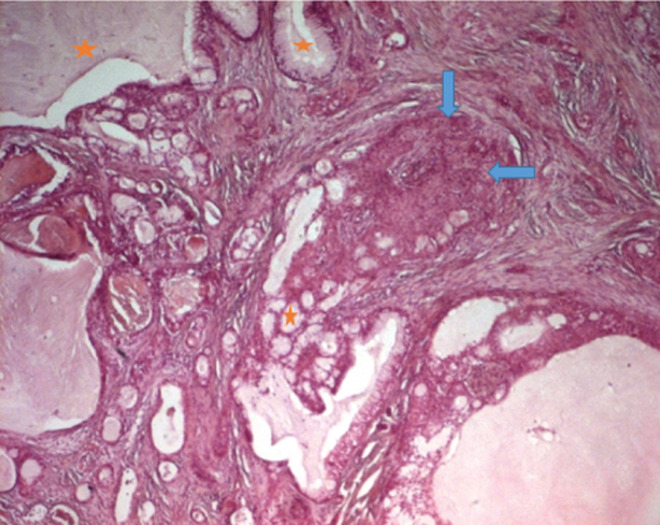
carcinome muco-épidermoïde: structures glandulaires (étoile orange), tapissées de cellules mucosécrétantes, associées à des massifs faits de cellules intermédiaires (flèche bleue horizontale) (HE x 100)

Les limites d´exérèses passaient en tissu tumoral, dans l´un des deux cas traités chirurgicalement et il n´y avait pas de métastase ganglionnaire. L´étude en IHC montrait un marquage positif de la protéine 63 (P63), l´actine muscle lisse (AML) la protéine S100 (PS100), le CD117 (Figure 5). Le marquage était négatif pour la chromogranine (CgA), la synaptophysine (Syn) et pour l´antigène carcino embryonnaire (ACE). Le Ki67 était estimé à 1%. Les deux tumeurs traitées chirurgicalement étaient classées p T2 N0 dans un cas et p T2 N1 dans l´autre selon la classification OMS 2015. Pour le carcinome muco-épidermoïde, les six cas étaient de bas grade de malignité, la tumeur était assez bien limitée, d´aspect biphasique, formée de structures glandulaires, tapissées de cellules mucosécretantes, associées à des massifs faits de cellules intermédiaires et de cellules d´allure malpighienne; les atypies étaient discrètes et les mitoses étaient rares. Le stroma était scléro-hyalin dans cinq cas et inflammatoire dans un seul cas (Figure 6).

Il n´y avait aucun cas de métastase ganglionnaire et les limites chirurgicales étaient tumorales dans un cas. Des lésions de fibrose pulmonaire, de dilatation de bronche, de bronchopneumonie et d´emphysème pulmonaire étaient notés chacun dans un cas. L´étude en IHC montrait une expression intense de la cytokéartine 7 (CK7), de la Vimentine (Vim) et focalement de l´antigène épithélial membranaire (EMA). Pour les cinq cas traités chirurgicalement, les tumeurs étaient classées en pT2N0 dans quatre cas et p T1N0 dans un cas selon la classification OMS 2015. En plus de la prise en charge chirurgicale, deux cas avec un CAK et un CME, non résécable non métastatique avait eu une chimiothérapie néo-adjuvante à base d´adriamycine à la dose de 40mg/m^2^ et cisplatine à la dose de 80mg/m^2^ avec un total de 6 cures, en association avec la radiothérapie. Trois cas dont deux cas de CAK et un cas de CME avaient eu une radiothérapie adjuvante. Deux patients avec un CME et un CAK ont une chimiothérapie palliative. La moyenne de survie globale était de 37.8 mois avec des extrêmes de 6 à 72 mois. Un patient était décédé après 24 mois du diagnostic. Six patients étaient vivants en rémission avec un recul qui variait de 24 à 72 mois. Deux patients étaient vivants en progression. Un patient était perdu de vue en évolutivité. Le Tableau 3 est un tableau comparatif entre le CME et le CAK.

**Tableau 3:** tableau comparative entre carcinome muco-épidermoïde (CME) et carcinome adénoïde kystique (CAK)

Type histologique	CME	CAK
**Clinique :**		
Age moyen	55.7 mois	41.8 mois
Sex ratio	1/3	½
Symptomatologie la plus fréquente	Douleur basithoracique + hémoptysie	Douleur basithoracique + hémoptysie
	3 mois et 6 mois	1 mois à 3 ans
Délai moyen entre le début des symptômes et la confirmation du diagnostic	3 OMS 1	2 OMS1
	3 OMS 2	1 OMS 2
Examen physique		1 OMS 3
Radiographie thoracique	3 opacités para cardiaques	3 opacités périphériques
	1 atélectasie + emphysème	1 opacité rétractile
	1 opacité rétractile	
	1 opacité périphérique	
**Scanner thoracique**		
	1 siège hilaire	3 intéressant la bronche lobaire
	3 intéressant la bronche souche	1 intéressant la bronche souche
	1 intéressant la bronche lobaire	
Stade tumoral cTNM 8^ème^ édition	3 stade I	3 stade III
	2 stade II	1 stade IV
	1 stade III	
Chirurgie (7/10 cas) :	5 cas	2 cas
**Macroscopie :**		
Taille moyenne	4.5cm	3cm
Aspect	Tumeur bourgeonnante et nodulaire, couleur beige ou blanc grisâtre de consistance ferme. Foyers hémorragiques dans un cas. A la coupe aspect microkystique et mucoide.	Tumeur bourgeonnante et nodulaire couleur beige ou blanc grisâtre de consistance ferme.
**Histologie :**	-Prolifération tumorale bien limitée, d’aspect biphasique, formée de structures glandulaires, tapissées de cellules mucosécretantes, associées à des massifs faits de cellules intermédiaires et de cellules d’allure malpighienne. Les atypies étaient discrètes et les mitoses étaient rares. -Le stroma était scléro-hyalin dans cinq cas et inflammatoire dans un seul cas. -Les limites chirurgicales étaient tumorales dans un cas. -Absence de métastase ganglionnaire.	-Prolifération tumorale organisée en massifs cribriformes ou tube à contenu éosinophile, positif avec la coloration de l’acide périodique de Schiff (PAS). Les cellules étaient monomorphes, cubiques ou arrondies, à cytoplasme parfois clair et réduit. Les atypies étaient discrètes et les mitoses étaient peu fréquentes. -Les engainements périnerveux étaient fréquemment notés. -Une infiltration de la trachée était notée dans un cas. -Les limites d’exérèses passaient en tissu tumoral, dans l’un des deux cas. - Absence de métastase ganglionnaire.
Stade p TNM (8 éme édition) :	pT2N0 (4 cas) pT1N0 (1 cas)	
**Immunohistochimie :**	-Intense positivité CK7 et vimentine. -Focal positivité EMA.	-Positivité du P63, AML, PS100, CD117 positif. -Négativité de la chromogranine A la synaptophysine et ACE. -Ki67 = 1%.
**Traitement néoadjuvant et adjuvant :**	Chimiothérapie néoadjuvant (1 cas): cisplatine +adriamycine Radiothérapie externe (1 cas) Chimiothérapie palliative (1 cas)	Chimiothérapie néoadjuvant (1 cas): cisplatine +adriamycine Radiothérapie externe (2 cas) Chimiothérapie palliative (1 cas)
**Evolution :**		
Nombre de patients décédés	1	0
Nombre de patients vivants en rémission.	5	1
Nombre de patients vivants en évolutivité	0	2
Nombre de patients PDV évolutivité	0	1

## Discussion

Les TMPGS représentent une entité hétérogène, qui associe plusieurs tumeurs, qui naissent à partir des glandes sous-muqueuses trachéo-bronchiques. Ces tumeurs sont observées habituellement au niveau des glandes salivaires peuvent être observées exceptionnellement ailleurs. Ce groupe de tumeur comporte : le CAK qui représente 0,09 à 0,2% de tous les cancers pulmonaires, le CME représentant 0.1 à 0.2% de tous les cancers pulmonaires, le CEM avec seulement 30 cas rapportés à l´échelle mondiale selon les données de la littérature, et le L´âge moyen de survenue des TMPGS pulmonaires se situe entre la quatrième et la cinquième décennie avec des extrêmes variant de 6 à 69 ans [1-4] et une fréquence plus élevée chez les sujets jeunes de moins de 30 ans pour le CME [5]. Le sexe ratio variait selon le sous-type histologique. Il existe une légère prédominance masculine pour le CAK, alors que le CME et le CEM atteignent aussi bien les hommes que les femmes [1-4]. Aucune relation avec le tabagisme ou autre facteur de risque n´a été démontrée jusqu´à présent dans la littérature [1]. Les circonstances de découverte sont variables, il s´agit le plus souvent d´une toux persistante. L´auscultation pulmonaire peut montrer une diminution du murmure vésiculaire des râles ronflants, un syndrome de condensation [1-4]. A la fibroscopie bronchique la tumeur présente un aspect bourgeonnant, souvent polypoïde ou lobulé, richement vascularisée et une obstruction est souvent retrouvée [1-4]. La radiographie du thorax montre une opacité bien limitée solitaire nodulaire de localisation endobronchique. Celle-ci peut être associée à une atélectasie, un collapsus, ou à un syndrome bronchique. La tomodensitométrie permet de mieux préciser le siège de la lésion, qui est généralement endobronchique, centrale, l´aspect, bien limité, lobulé, ou nodulaire, parfois obstructif, les lésions associées telles que l´atélectasie et l´étendue et l´extension de la tumeur dans le médiastin, ainsi que dans le reste de l´organisme [6,7]. Le diagnostic repose sur l´étude anatomo pathologique. Pour le carcinome adénoïde kystique, il est posé devant une tumeur ayant un double contingent de cellules épithéliales et myoépithéliales, assez monomorphes et peu atypiques, montrant une architecture cribriforme d´aspect cylindromateux et microkystiques occupés par un matériel hyalin mucoïde et basophile, soit une architecture tubulaire ou solides. Le stroma est généralement fibro-hyalin. Les engainements périnerveux sont fréquents. La nécrose est exceptionnelle et les métastases ganglionnaires sont peu fréquentes [8-10]. La tumeur est très infiltrante, d´où l´importance de l´examen extemporané dans la vérification de l´état des limites d´exérèse. A l´étude immunohistochimique, Les cellules tumorales expriment la P63, l´AML, la PS100 et le CD117 (c-kit). Alors que le facteur de transcription thyroïdien (TTF1) et le CD56 sont négatifs [8-10].

A l´étude cytogénétique et en biologie moléculaire, une perte de l´hétérozygotie du chromosome 3p14 et du 9p est décrite, mais aucune mutation de l´*epidermal growth factor* (EGFR) et du *Kirsten rat sarcoma viral oncogene homologue* KRAS n´a été rapportée. Le réarrangement MYB, correspondant à une translocation t (6; 9) (q22-23; p23-24) est spécifique du CAK, il peut être utile en cas de doute diagnostique avec un adénocarcinome [8,10]. Concernant le carcinome muco-épidermoïde, le diagnostic histologique est posé devant une prolifération faite de cellules mucosécrétantes, de cellules intermédiaires, et de cellules malpighiennes. Les cellules s´agencent en lobules souvent kystisés et séparés par des travées conjonctives fibreuses plus ou moins denses [11]. Il existe deux grades histologiques selon l´abandance des cellules malpighiennes, la présence de nécrose, d´atypies cyto-nucléaires et l´activité mitotique [11]. Dans notre étude, les cas de CME étaient tous de bas grade. A l´étude immunohistochimique, les cellules expriment la CK7 et la CK5/6, et n´expriment pas le TTF1 [12]. Sur le plan génétique, Le CME est associé à une translocation, impliquant le muco-épidermoid carcinoma translocated1 (MECT1), au niveau 19p13 et le mastermind-like gène family2 (MAML2), au niveau 11q21: t (11; 19) (q21; p13). L´identification du gène de fusion MCT1-MAML2 par réaction en chaîne par polymérase (PCR) ou par hybridation in situ fluorescente (FISH), est considérée comme pathognomonique pour cette entité. Aucune mutation du gène de l´EGFR n´a été retrouvée [12]. Le diagnostic du carcinome épithélial-myoépithélial est posé devant une tumeur faite de structures canalaires composée de deux couches de cellules : une couche interne formée de cellules épithéliales et une couche externe formée de cellules myoépithéliales à cytoplasme clair. Les atypies sont légères, l´activité mitotique est variable mais généralement les mitoses sont rares. La nécrose et l´engainement périnerveux sont exceptionnels. A l´étude immunohistochimique, les cellules de la couche interne de cellules épithéliales expriment les CK, l´EMA, l´ACE, l´expression est variable pour le TTF1 et pour la couche externe, les cellules expriment les CK, la P63, la PS100 et l´AML, le TTF1 est négatif [13]. Sur le plan moléculaire, des altérations de la protéine P53 jouent un rôle dans le développement de la tumeur, par contre, aucune mutation du gène KRAS et de l´EGFR n´a pas été retrouvée [14].

Enfin, le carcinome ex-adénome pléomorphe correspond à une transformation carcinomateuse d´un adénome pléomorphe, son diagnostic est posé sur la présence d´atypies marquées et diffuses, de mitoses nombreuses et atypiques, de nécrose tumorale, d´un envahissement capsulaire et du tissu de voisinage, des emboles vasculaires et des engainements nerveux [15]. L´étude immunohistochimique montre une positivité pour la pancytokératine, la CK7, l´AML, et la P63 et une négativité pour le TTF1 [15]. A l´étude cytogénétique, des anomalies du chromosome 8q21, des réarrangements PLAG1 ainsi que des amplifications HMGA sont retrouvés [15]. Le traitement des TMPGS pulmonaires est essentiellement chirurgical, pour le carcinome adénoïde kystique, la résection complète est souvent difficile à obtenir, en rapport avec le caractère infiltrant de la tumeur, le long des voies aériennes ; la radiothérapie est indiquée en cas de tumeur inopérable, ou en cas d´exérèse incomplète, pour contrôler les lésions tumorales résiduelles et les récidives [16]. Des cas de tumeurs inopérables ont été traités par radiothérapie en association avec la chimiothérapie hebdomadaire à base de carboplatine et paclitaxel, avec une bonne tolérance et une réponse objective radiologique [16]. La chimiothérapie adjuvante est indiquée dans les CME de haut grade. En néo- adjuvant, elle a montré son efficacité dans ce type de tumeurs de stade 4. L´association carboplatine-paclitaxel peut être une option dans le traitement de ces tumeurs. La combinaison cisplatine-docetaxel a également donné une bonne réponse radiologique [17]. En ce qui concerne le pronostique, les récidives locales sont fréquentes et tardives pour le carcinome adénoïde kystique, il est donc nécessaire de programmer une surveillance à long terme, sur 10 à 15 ans avant d´affirmer la guérison. Les métastases à distance sont rares et également tardives. La survie à 1 an et 10 ans est de 85% et 39%, respectivement. La forme solide est un facteur de mauvais pronostic [9]. De nombreux travaux récents ont montré que l´expression des marqueurs comme la P53, et le Ki67 est associée à un mauvais pronostic, alors que l´absence d´expression du CD117 est un sujet de controverse [8]. Les CME de haut grade ont une évolution défavorable et sont particulièrement agressifs avec une extension trans-bronchique rapide vers le parenchyme pulmonaire adjacent et les voies lymphatiques, entraînant des métastases ganglionnaires, osseuses, surrénaliennes, cérébrales et cutanées ; contrairement aux CME de bas grade qui sont de bon pronostic avec un risque métastatique de 5%. Les facteurs histologiques de mauvais pronostic sont représentés par une proportion faible de cellules mucosécrétantes, un engainement péri-nerveux, l´index mitotique supérieur à 4 mitoses pour 10 champs, la présence de secteurs de nécrose ou anaplasiques et une proportion d´espaces kystiques inférieure à 20% de la surface tumorale. Tous ces facteurs sont associés à une faible survie à 5 ans et à une fréquence élevée de rechutes post opératoire. Par ailleurs, la fusion MECT1-MAML2 est 0corrélée à une meilleure survie [18]. Le carcinome épithélial-myoépithélial a une évolution favorable; les récidives et les métastases sont exceptionnelles [19]. Le carcinome ex-adénome pléomorphe est une tumeur agressive, de mauvais pronostics, associés à une fréquence élevée de récidives locales et métastatiques [15].

**Limites de notre étude**: a) le refus de certaines patientes à participer à l'étude; b) le manque d´information dans les dossiers médicaux a été une difficulté lors de la collecte des résultats; c) le caractère rétrospectif de cette étude.

## Conclusion

Les tumeurs malignes primitives de type glandes salivaire pulmonaires forment un groupe hétérogène de tumeurs peu agressives mais associées à une fréquence élevée de récidive et de métastases tardives imposant une surveillance à long terme, il est important de les distinguer des autres tumeurs broncho-pulmonaires, dont elles ne partagent ni la biologie ni le traitement ni l´évolution.

### Etat des connaissances sur le sujet

- Les tumeurs des glandes salivaires se voient fréquemment au niveau de la cavité buccale;
- Ces tumeurs sont le plus souvent bénignes;
- Les tumeurs pulmonaires sont le souvent des carcinomes épidermoïdes et des adénocarcinomes.

### Contribution de notre étude à la connaissance

- Devant la rareté des tumeurs des glandes salivaires au niveau du poumon, on va détailler les aspects clinico-pathologiques de 10 cas de tumeurs malignes primitives de type glandes salivaire du poumon colligés sur une période de 32 ans;
- Décrire les particularités pronostiques et thérapeutiques de cette rare entité;
- Faire une revue de littérature de cette entité peu connue par les pneumologues et oncologues.
